# Applications of Artificial Intelligence in Climate-Resilient Smart-Crop Breeding

**DOI:** 10.3390/ijms231911156

**Published:** 2022-09-22

**Authors:** Muhammad Hafeez Ullah Khan, Shoudong Wang, Jun Wang, Sunny Ahmar, Sumbul Saeed, Shahid Ullah Khan, Xiaogang Xu, Hongyang Chen, Javaid Akhter Bhat, Xianzhong Feng

**Affiliations:** 1 Key Laboratory of Soybean Molecular Design Breeding, Northeast Institute of Geography and Agroecology, Chinese Academy of Sciences, Changchun 130102, China; 2 Zhejiang Lab, Hangzhou 310012, China; 3 Institute of Biology, Biotechnology and Environmental Protection, Faculty of Natural Sciences, University of Silesia, Jagiellonska 28, 40-032 Katowice, Poland; 4 National Key Laboratory of Crop Genetic Improvement, Huazhong Agricultural University, Wuhan 430070, China

**Keywords:** artificial intelligence (AI), crop breeding, genomics, phenomics, envirotyping, big data

## Abstract

Recently, Artificial intelligence (AI) has emerged as a revolutionary field, providing a great opportunity in shaping modern crop breeding, and is extensively used indoors for plant science. Advances in crop phenomics, enviromics, together with the other “omics” approaches are paving ways for elucidating the detailed complex biological mechanisms that motivate crop functions in response to environmental trepidations. These “omics” approaches have provided plant researchers with precise tools to evaluate the important agronomic traits for larger-sized germplasm at a reduced time interval in the early growth stages. However, the big data and the complex relationships within impede the understanding of the complex mechanisms behind genes driving the agronomic-trait formations. AI brings huge computational power and many new tools and strategies for future breeding. The present review will encompass how applications of AI technology, utilized for current breeding practice, assist to solve the problem in high-throughput phenotyping and gene functional analysis, and how advances in AI technologies bring new opportunities for future breeding, to make envirotyping data widely utilized in breeding. Furthermore, in the current breeding methods, linking genotype to phenotype remains a massive challenge and impedes the optimal application of high-throughput field phenotyping, genomics, and enviromics. In this review, we elaborate on how AI will be the preferred tool to increase the accuracy in high-throughput crop phenotyping, genotyping, and envirotyping data; moreover, we explore the developing approaches and challenges for multiomics big computing data integration. Therefore, the integration of AI with “omics” tools can allow rapid gene identification and eventually accelerate crop-improvement programs.

## 1. Introduction

Plant breeding is a time-honored tradition that continues the process of developing improved plant cultivars, which dates back to the dawn of agriculture. Humans began to discern degrees of excellence among the plants in their fields soon after the initial domestications of cereal grains, from which they stored the seeds of the best to grow new crops. Early plant-breeding technologies were the forerunners to such rudimentary selection strategies [1]. People from all across the world explored and cultivated nearly 7000 varieties of food plants during Breeding 1.0, which began 10–12 thousand years ago [2]. Breeding stage 2.0 started in the late nineteenth and early twentieth centuries when an inbreeding depression was discovered. During this time, many advances in plant breeding were made in the science of breeding itself, such as replicated field trials, controlled crossings, statistical analyses, formal experimental designs, hybrid breeding, pedigree-based estimates of breeding values, and precise yield measurement at scale (e.g., with multirow combines) [3]. Breeding 3.0 reached to about 30 years ago, when molecular markers and genomic data began to supplement phenotypic data [4]. Breeding 4.0 is rapidly approaching, with huge omics data and the rapid progress of informatic technologies [5]. Since the beginning of the plant breeding process, starting from crop domestication, new approaches mediated by different technological revolutions are being enriched in the science continuously to increase the pace, accuracy, and precision in plant breeding [6]. In the past decade, this science has already brought the green revolution by developing semi-dwarf, nutrient-responsive, and hybrid cultivars [7]. However, considering the population growth, decreasing arable land, and climate changes, these have demanded more precise, high-throughput approaches that can mediate to develop crop cultivars at a greater pace, with higher accuracy, and precision. In this regard, the field of artificial intelligence has recently emerged, which has been suggested to possess extraordinary potential to assist in breeding climate-resilient smart crops. Climate-resilient crops maintain or increase crop yields and quality under various climatic and environmental changes, and possess the ability to resist multiple biotic and abiotic stresses. To this end, climate-resilient smart crops will allow crops to address the interlinked challenges of food security and climate change [8].

The goal of artificial intelligence (AI) is to replicate some features of human intelligence using technology [9]. This discipline can be defined as a set of studies and techniques dealing with the computer science and mathematical aspects of statistical modeling, with significant economic and social implications, and the goal of developing technological systems capable of solving problems and performing tasks or duties normally performed by the human mind [10]. The increased interest in AI in the breeding world is due to the technological maturity attained, i.e., the ability to analyze large amounts of data in a short period of time to reveal unexpected linkages. The AI approach from a breeding standpoint, allows individuals to systematize information that is typically already available on the market in a disaggregated form, transforming data into breeding decisions, and thus only considering those tools that are useful to facilitate decision-making processes in crop breeding.

This paper presents how AI technologies help to solve problems of high-throughput phenotyping and gene functional analysis in current breeding practices, as well as tackle the current massive-data processing bottlenecks in both phenotyping and genotyping, and bring new avenues for future breeding to make envirotyping data extensively used in breeding.

## 2. AI Technologies Benefiting Crop Breeding

Artificial intelligence uses computers and technology to simulate the human mind’s problem-solving and decision-making skills [11]. AI, often known as machine intelligence, is an area of computer science that focuses on developing and managing technology, which can learn to make decisions and carry out activities independently without the need for human effort [12]. AI is a broad term that encompasses a wide range of technologies; it is a catch-all word for any software or hardware component that helps with machine learning, computer vision, natural language comprehension, and natural language processing (NLP) [13]. Traditional complementary metal-oxide-semiconductor (CMOS) hardware and the same fundamental computational processes that drive traditional software are used in today’s AI [14]. AI is the most rapidly emerging technology in computer science in today’s digital world, and it creates intelligent computers that replicate the intellect of the human mind [15]. For instance, the deep neural network (DNN), artificial neural network (ANN), random forest (RF), and support vector machine (SVM) are a few examples of machine-learning algorithms, as well as advanced hi-tech equipment such as the internet of things (IoT) [16]. AI is a fascinating hi-tech system that provides an endless opportunity as far as its agricultural applications are considered; hence, this opens up new frontiers for digital breeding [17]. Future AI generations are projected to inspire new sorts of brain-inspired circuits and architectures capable of making data-driven judgments faster and more precisely than humans can [18]. Furthermore, artificial intelligence, big data, machine learning, and data analytics are all terms that appear often in current academic and corporate writings that deal with data [19].

Big data, machine learning, and AI are some of the terms used to characterize modern computer processes [20]. Big data is concerned with the use of huge data of diverse types and complex structures that cannot be handled well when analyzed through classical approaches [21]. In this context, the AI trains a computer to perform jobs that are beyond human efforts, especially by considering the time and labor involved, and which are typically involved in decision-making in a variety of situations [22]. Machine learning (ML) is a branch of AI in which computers discover relationships from massive training datasets. For environment and weather applications, a simple definition is: firstly, big data involves the collection of meteorological or Earth System-related measurements, as well as high spatial and temporal resolution Earth System model (ESM) outputs for analysis; secondly, ML is the refining or discovery of new linkages between locations, times, and quantities in the datasets (e.g., where sea surface temperature features aid the weather prediction for months over land regions); thirdly, AI is a means of providing automatic warnings and guidance to society in the event of oncoming weather extremes, based on the links discovered by machine learning [23]. The current ease for application of ML methods due to improved computing capabilities is aided in part by the unique usage of computer graphics processing units (GPUs), with GPU speed improving at a quicker rate than ordinary central processing units [24]. This is an innovative use of computer memory to make calculations both more efficient and considerably closer to the data storage location [23]. The main emphasis of employing AI in breeding is that it complements the work of the breeder by guaranteeing continuous farm monitoring. Indeed, with the automation of farms and the generalization of data, breeders may dedicate more time to higher-value jobs by spending less time in their buildings. AI saves time in data identification and processing, which is of considerable benefit. Breeders and technical advisors acquire confidence and reactivity, allowing them to act when it is most appropriate [25].

AI technology has been used to accelerate the process of breeding new plant varieties, such as high-throughput genomics and phenomics to advanced breeding [15,26,27,28]. Increasingly, ML methodology has been used in genomic prediction, genomic selection, and marker-assisted selection [27,28]. Many agricultural companies such as Monsanto and John Deere have already invested hundreds of millions of dollars to develop such technologies that can utilize extensive data on soil type, seed variety, and weather to help farmers reduce costs and enhance yields [29]. Many of the same data sources, such as weather forecasts and Google Maps, are used to fuel both of their businesses. In addition, they may access farm equipment data that are wirelessly sent to the cloud [30]. As part of a precision-farming experiment in Romania, companies like Nippon Electric Company, Limited (NEC; headquartered in Minato, Tokyo, Japan) and Dacom (headquarter in Santa Clara, USA) employed environmental sensors and huge data analytics tools to increase yields. The use of current technologies and information systems enhances the overall productivity of agriculture [31]. Due to the agricultural data sets’ complexity, novel architecture and frameworks, algorithms, as well as the analytics face several obstacles in extracting the value and hidden information from this data [32]. The recent research on AI tools, including ML, deep learning, and predictive analysis intended toward increasing the planning, learning, reasoning, thinking, and action-taking abilities [33]. Plant Breeders are developing systems to aid in a better understanding of plant behavior under a variety of climatic situations [34]. Summit, the world’s most powerful supercomputer, was recently unveiled with the potential to hold 27,000 GPUs, paving the way for a bright future. AI has the potential to be a game-changer in the near future for bringing an agricultural revolution and global food security [35].

## 3. AI Technologies Overcoming the Phenomics Bottlenecks

Plant phenomics has advanced rapidly in recent years providing the scope for precision breeding. This progress can be ascribed to an increase in the invention and availability of new technologies that allow for high-throughput phenotyping of complex plant features. In recent years, the use of AI in a variety of scientific fields has exploded. AI features, viz., computer vision, ML, and deep learning have been effectively integrated into non-invasive imaging procedures. Through the use of ML for robust picture analysis, this integration is steadily enhancing the efficiency of data gathering and analysis. Furthermore, AI has aided the development of software and tools for data gathering and management in field phenotyping. These include open-source devices and platforms that allow for community-driven research and data sharing, providing the enormous amounts of data needed for reliable phenotyping research [36] AI is used in three critical components of phenomic data management: algorithms and programs to convert sensory data into phenotypic information; model development to understand genotype-phenotype relationships with environmental interactions; database management to allow information and resources to be shared [36].

Experiments involving repeated trials in diverse conditions (considering the statistical need for an unbiased estimation) are required in order to screen plants for desirable features (such as grain size, abiotic stress tolerance, product quality, or yield potential). The measuring of individual plants in controlled conditions has been the subject of considerable phenotyping discussion; however, plant development under open-air circumstances is not accurately represented in controlled environments [37]. These things considered, a large gap has been seen practically regarding the performance of plants from lab-to-field [7]. The ongoing integration of AI into various technologies promises a development toward smarter, faster, and lower-cost solutions. In comparison to other imaging techniques, the integration of AI into the data management pipeline of tomography and thermography is on a smaller scale in the area of phenotyping image data analysis. Deep learning has been successfully used in the analysis of composite materials [38]; therefore, its application in the data analysis of these approaches is promising. Despite the fact that field phenotyping is a practical need in the crop breeding, still the high-throughput phenotyping under field conditions lags behind the indoor phenotypic facilities currently available. Thus, it needs more effort to develop such facilities to explore the practical aspects of phenomics. To increase their accuracy, AI technologies require a significant amount of data from numerous sources. This opens up the possibility of investing more in the customization of current technologies for field-data collecting, and the use of already available AI adaptable technology, such as smartphones, to boost the number and quality of data collected [39]. Smartphones have become common consumer items and the ease with which their sensors may be used suggests their great application in agriculture [36]. Advanced signal processing on smartphones must contend with constraints such as low battery life, limited computational power, and limited bandwidth [36]. The use of citizen science in data collecting alongside professional researchers has the potential to increase the amount of data collected [39]. The main purpose of using these approaches and technology is to offer the infrastructure for tracking how plants progress during the growing season and to make the data analysis, management, and use of results via AI methods easier [40].

Recent studies have showed that the phenotyping of crops through AI shows an improvement in crop phenotyping and predictions [41,42,43,44,45,46,47]. For example, Selvaraj et al. (2020) [41] reported that ML algorithms, viz., k-Nearest Neighbours (kNN), RF, and SVM revealed the best performance for root yield prediction in the cassava (*Manihot esculenta* Crantz), with the highest accuracy of R^2^ = 0.67, 0.66, and 0.64, respectively. Moreover, AI-assisted high-throughput phenotyping systems have been successfully applied to: wheat and maize to identify the plant growth stage [42] and plant image segmentation [43]; oilseed crops for semantic segmentation of the crops and weeds [44]; the phenotyping of disease resistance of crops [45]; improvement of plant productivity [46,47].

## 4. Exploring the Potential of AI in Gene Function Analysis

The rapid development of high-throughput technologies in biological sciences has resulted in the generation of massive data in recent decades. Disciplines that attempt to collect and analyze enormous volumes of biological data are often referred to as “omics”, which is used to indicate the total quantity of DNA contained in each cell of an organism, with an additional flavour of openness to big challenges [47]. “Omics” data has become too large and complicated to be analyzed visually or by using statistical correlations. This has incited the use of so-called Machine Intelligence or AI which manages large amounts of data that are insurmountable for human minds, while extracting information that goes beyond our current understanding of the system under investigation and, most importantly, improving automatically based on the training data [48].

AI is already being used extensively in plant genomics and also possesses more future applications for in-depth genome exploration. A number of ML tools and algorithms are available for different kinds of bioinformatics analysis, such as protein-coding gene identification, *cis*-regulatory element identification, gene expression, subcellular location, protein-protein interaction, gene ontology, metabolic pathways, phenotypes, and genomic prediction (as reviewed by Mahood et al. (2020) [49]). In the not-too-distant future, AI is likely to be used to address a variety of plant science genomics concerns.AI algorithms might potentially be used to address comparative genomic investigations or information transfer from a model plant to a crop of interest [50]. DeepBind [51] and DeepSEA [52] are two models that have been created in recent years to predict and analyze genetic features [26]. Various sorts of expressions or sequencing data analysis can be thought of, with the goal of predicting gene functions or the differential effects of gene expression on a trait [53].

Although a significant amount of genomic data was produced as a result of the fruitful breakthroughs of high-throughput sequencing technology, the enormous amount of data generated creates a huge problem for storage and examination of the data [26]. The AI technology of bioinformatics enables the measurement of simultaneous expressions of a large number of genes, or even each and every gene that is included in the genome under a wide range of situations [54,55]. All of this combines to give biologists a more “relevant” representation of their data and the ability to integrate it, which enables them to examine their genomic data, test and confirm their assumptions throughout the experimental cycle, and ultimately improve their research [56,57].

## 5. Linking of Crop Genome to Phenome with AI

**Figure 1:**
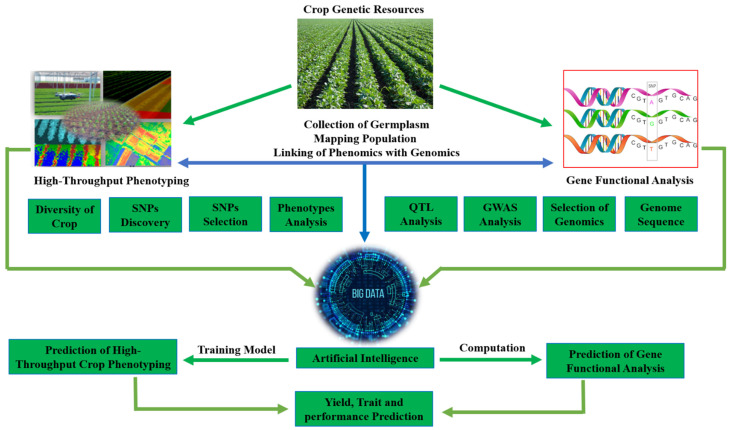
Artificial Intelligence used as a powerful tool for the prediction of high-throughput crop phenotyping and gene functional analysis in modern crop breeding. The high-throughput phenotypic and genotypic data were collected from large crop germplasm and breeding populations. The massive comprehensive database could integrate various resources with AI technology, such as phenotypic diversity of crops, SNPs polymorphisms, QTL analysis, GWAS analysis, genomics selection, and genome sequence. AI technologies are applied to predict the crop phenotype with whole genome prediction, the novel breeding strategies are produced through AI related to computation and training models.

Currently, modern breeding approaches are focused on linking the genotype with the crop phenotype accurately and precisely. In advanced breeding, linking the whole of the genome information to high-throughput phenotypes remains a massive challenge, and is impeding the optimal application of field phenotyping and omics [15]. Germplasm collection and mapping populations can efficiently differentiate the phenomics and genomics data through AI. Crop diversity, single nucleotide polymorphisms (SNPs) detection and selection, quantitative trait loci (QTL) analysis, genome-wide association study (GWAS) analysis, and genomic selection and sequences generate a large amount of data; AI can evaluate and link the phenomics and genomics data from these big data to improve the breeding approaches. AI related to a computation and training model can predict the gene functional analysis and high-throughput crop phenotyping and also predict the performance of yield and traits of the crop [46,47,50,58,59]. Therefore, the integration of AI with phenomics and genomics tools can allow for rapid gene identification associated with the crop phenotypes that eventually accelerate crop improvement programs. In Figure 1, we summarize how to apply AI technology to link high-throughput genomics and phenomics, which can result in the production of better breeding strategies.

Research on crop genomics is not only understanding the molecular mechanisms of phenotypes but also using technical data and bioinformatics techniques to analyse and understand the molecular mechanisms behind phenotypes [60]. To date, AI is a fascinating approach to bringing out these tasks inevitably [61]. AI approaches provide the platform to analyze huge, various, and useless datasets such as the generation of genome sequencing/photo imaging over conventional analytical strategies [15,62]. Recently, the AI approach has been explicitly employed in varied research fields of phenomics and genomics, such as: analysing genome assembly and genome-specific algorithms [26]; broad-range data analysis to mitigate multiplex biological complications in metabolomics, proteomics, genomics, transcriptomics, as well as systematic biology [62,63]; interpretation of gene expression cascades [64,65]; identification of significant SNPs in polyploid plants [66]; high-throughput crop stress phenotyping [41,67].

Scientists have employed AI and its developed models to modulate the flow of information from generic DNA to genetic-based phenotypes, to investigate the potential variants in natural populations [49]. More specifically, for breeders, AI will assist the further investigation of genetic loci to facilitate the agricultural output by triggering the genome algorithms and allowing high-throughput crop phenotyping in quantitative traits for open-field and controlled environments [49,68]. Additionally, AI can be cohesively combined with bioinformatics and genome sequencing analysis to interpret various molecular repertories such as transcription factor binding sites [69], long non-coding RNAs (lncRNAs) [70], microRNA (miRNAs), epistatic modifications, coding genes, targeted polyadenylation sites [71], as well as *cis*-regulatory elements (CREs) [49,72].

Various crop databases insert a huge amount of heterogeneous-related phenotypic and genotypic data (big data) recently providing insight into potential resources for breeders to untangle novel trait-identified candidate genes [73]. Luckily, AI provides a novel benchmark summary for analytical and computational methods for the integrated analysis of such enormous datasets based on the big-data spectrum [49,73]. In addition, employing AI to conclude the interrelations between candidate genes and CREs is a novel approach for categorizing and identifying previously unknown genes for significant crop improvements [74]. Furthermore, AI strategies have more potential for interpretation of the crop yield, variation in climatic assessment, high-throughput crop stress phenotyping, climate temperature, ultraviolent (UV) radiation, wind, and hail [26,73,75]. The role of AI is becoming more and more important in obtaining, analyzing, integrating, and managing genomic and phenomic data to increase agricultural climate resilience [76,77].

Next generation sequencing (NGS)-based genotyping methods have helped to improve gene-mapping resolution and gene identification and NGS-based genotyping for GWAS analysis has been used in crop improvement [68]. For example, in soybean, these kinds of studies have been widely used to identify genetic loci and candidate genes for seed weight [78], seed protein and oil contents [79], pod dehiscence [80], nitrogen fixation [81], soybean plant height and primary branches [81], agronomic traits [82], disease resistance [83], and tocopherol concentration [84]. Bulk segregant analysis (BSA) and its modified methodologies are currently used in many crops [85,86,87,88,89,90]. The NGS-based BSA is becoming a popular approach to identifying candidate genes for various traits, such as the soybean mosaic virus [91], charcoal rot resistance [92], flowering time [93], phytophthora resistance [94], and powdery-mildew resistance [95]. Recently, the deep-learning algorithm for BSA (DeepBSA) has been developed for QTL mapping and functional gene cloning in maize [96].

## 6. AI Making Envirotyping Data Accessible in Crop Breeding

Climate change has a great impact on the environment and crop production for the present and future. The concept of envirotyping is proposed as a third “typing” technology, accompanying phenotyping and genotyping to decode environmental influences on crop breeding [97]. Envirotyping plays a key role in crop modeling and the prediction of phenotypes through its efficient components, including the genotype-by-environment interaction (GEI), environmental signals, responsive genes, biotic and abiotic stresses, as well as integrative phenotyping [76]. Cortes et al. (2022) discuss the state of the emerging field of study known as “genome-environment associations”, which combines ecological climatic data with evolutionary genomics (GEAs) [98]. The authors advocate for the community to begin collecting genomic estimated adaptive values (GEAVs) for genomic prediction (GP) and multi-dimensional ML models in order to take polygenic evolutionary adaptation into consideration. Xu et al. (2022) recently proposed an integrated genomic-enviromics prediction breeding scheme using integrated multiomics information, big data technology, and artificial intelligence [99].

Climate change, as well as the global population and pathogen pressure, have raised serious alarm over worldwide food security. In the coming years, strategies need to be developed to maximize the limited resources and their utilization for crop breeding and land management [100]. Climate-smart crops and climate-smart soils have been adapted to the environment for more effective breeding programs, and breeders may use this knowledge to generate new smart crops for the new climate. Genomic technologies together with high-throughput phenotyping are providing researchers and farmers with the information required to guide and notify the breeding methods for climate-smart breeding [76]. AI plays a vital role in integrating and manipulating this fast collecting abundance of data by conducting association studies to identify genomic targets which are related to adaptive climate-resilient traits [101]. Cortes et al. (2021) also put forward a roadmap to use ML, GP, and multi-trait gene editing approaches to capture novel abiotic stress tolerance variations from wild crop relatives to utilize these variants for bread drought-tolerant crops [102]. Breeders can use these data to adjust crops to their environment and they can be introduced via advanced selection or genome editing techniques [15]. Genomic and phenomic data will need to be integrated into comprehensive clade-specific databases and platforms, as well as accessible tools that breeders may utilize to inform the selection of climate-adapted characteristics, to effectively translate research into the field.

**Figure 2:**
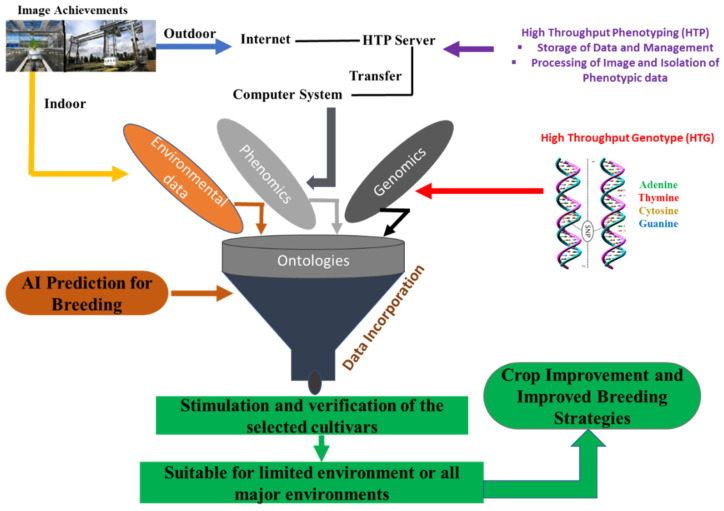
Integration and management of Genomics, Phenomics, and Enviromics data by artificial intelligence for crop-breeding improvement. The phenotypic data of crops are collected from both indoor and outdoor environments, the information of phenotypic, genotypic and environmental are combined together with AI technology. With mathematical modelling, logical deduction, and decision-making, the AI-assisted breeding system will simulate and verify the selected cultivars, whether it is suitable for cultivation in limited environments or all major environments.

Our proposed breeding scheme (Figure 2) integrates genotypic, phenotypic, and envirotypic information to improve efficacy. The phenotypic data of crop plants both for indoor and outdoor environments are collected by high-throughput robotic systems [101] and the phenotypic information from various environments will transfer to a high-throughput phenotyping (HTP) server via Internet. The multiple datasets will take the genotypic, phenotypic, and envirotypic information together, and the G × E interaction (GEI) will quantify by multiple environments. AI technology, particularly ML and deep learning (DL), is used in cultivar selection for specific or multiple major environments. This approach will enable us to make decisions about the selected cultivars, and whether it is suitable for cultivation in limited environments or all major environments.

## 7. Future Prospects of AI Breeding

In recent years, there has been significant growth in discussion regarding the importance of AI leading to debates about the applications of AI in the world. Plant breeding must be updated to take advantage of the digital revolution. Researchers and breeders must evaluate computer-generated suggestions against farmers’ demands to be successful in their future work. Many sectors throughout the world, including agriculture breeding, are benefiting from greater profitability and have high economic growth rates as a result of the introduction and successful deployment of AI technologies. Furthermore, AI will focus on developing novel, human-centered techniques, assessing the application of robotic technology to a variety of industries and businesses around the world. AI will also transform the way different companies around the world expand and compete by representing new manufacturing concepts that will result in breeding profitability. To take full advantage of such prospects, most firms throughout the world will need to be more involved in the creation of various AI methods, such as putting human aspects at the centre of the nucleus. They will also concentrate on developing a variety of responsible AI machines with moral and ethical ideals, which will lead to positive outcomes as well as to enable individuals to perform tasks they are familiar with. The development of various AI systems will assist the global agricultural breeding sector in assuming the availability of symbolic structures, such as reasoning ability and knowledge existence. Furthermore, when AI achieves intelligence comparable to or higher than that of humans, there will be concerns about societal and political change.

These and other instances suggest that AI holds promise for some genomics applications. What does all of this have to do with plant genomics? One observation is that large-scale datasets, which are required to train the AI applications listed above, are not currently accessible for plants. In the not-too-distant future, AI is likely to be used to address a variety of plant science genomic concerns. It is possible that one area of plant genomics that may be addressed is how to deal with several species (such as wheat, soybean, rice, maize, tomato, and oilseed rape) at the same time, intercropping is becoming important for covering crops of mixed species. AI algorithms might potentially be used to address comparative genomics investigations or information transfer from a model plant to a crop of interest.

Farmers and breeders will be able to feed the data into cloud-based AI applications via portable devices, drones, and agriculture-equipment platforms making AI applications more widely accessible. The phenomics and genomics data obtained with ML and DL are accurate, but not good enough to totally rely on the technology to speed up breeding, which is still a tough, time-consuming, and costly process. When examined in genomes, epigenomics, transcriptomics, proteomics, metabolomics, and phenomics still provide minimal information.

Furthermore, plant scientists are finding amazing answers to most of these problems, changing our emphasis from algorithmic performance to new farming models that could enable a new agricultural revolution that is better for both humankind and the environment. AI will efficiently revolutionize the “omics” approaches and breeding management with high-technology methods. AI will develop a large number of genotypes and phenotypes that must be screened using model-based envirotyping for a wide range of adaptive genotypes and phenotypes, and segregating material must be developed and advanced using speed-breeding management or fast-generation advancement to shorten the breeding cycle and improve genetic gain.

## Data Availability

No new data were created in this study. Data sharing is not applicable to this article.
